# Genome-Wide Identification and Evolutionary Analysis of Functional *BBM-like* Genes in Plant Species

**DOI:** 10.3390/genes15121614

**Published:** 2024-12-17

**Authors:** Zhengyuan Hong, Linghong Zhu, Chaolei Liu, Kejian Wang, Yuchun Rao, Hongwei Lu

**Affiliations:** 1 College of Life Sciences, Zhejiang Normal University, Jinhua 321004, China; 15325984343@163.com (Z.H.); zhulh0304@zjnu.edu.cn (L.Z.); 2 State Key Laboratory of Rice Biology and Breeding, China National Rice Research Institute, Chinese Academy of Agricultural Sciences, Hangzhou 310006, China; liuchaolei@caas.cn (C.L.); wangkejian@caas.cn (K.W.)

**Keywords:** phylogenetic analysis, collinearity analyses, duplication event, miRNA prediction, molecular docking, expression pattern

## Abstract

**Background/Objectives**: BABY BOOM (BBM), a transcription factor from the APETALA2 (AP2) protein family, plays a critical role in somatic embryo induction and apomixis. *BBM* has now been widely applied to induce apomixis or enhance plant transformation and regeneration efficiency through overexpression or ectopic expression. However, the structural and functional evolutionary history of *BBM* genes in plants is still not well understood. **Methods**: The protein sequences of 10 selected plant species were used to locate the branch of BBM-Like by key domain identification and phylogenetic tree construction. The identified *BBML* genes were used for further conserved motif identification, gene structural analysis, miRNA binding site prediction, cis-acting element prediction, collinear analysis, protein–protein interaction network construction, three-dimensional structure modeling, molecular docking, and expression pattern analysis. **Results**: A total of 24 BBML proteins were identified from 10 representative plant species. Phylogenetic relationship analysis displayed that BBML proteins from eudicots and monocots were divided into two clusters, with monocots exhibiting a higher number of BBMLs. Gene duplication events indicated that whole genome/segmental duplication were the primary drivers of *BBML* genes’ evolution in the tested species, with purifying selection playing a key role during evolution processes. Comparative analysis of motif, domains, and gene structures revealed that most BBMLs were highly evolutionarily conserved. The expression patterns of *BBML* genes revealed significant tissue specificity, particularly in the root and embryo. We also constructed protein–protein interaction networks and molecular docking models to identify functional pathways and key amino acid residues of BBML proteins. The functions of BBMLs may differ between monocots and eudicots, as suggested by the functional enrichment of interacting proteins. **Conclusions**: Our research delved into the molecular mechanism, evolutionary relationships, functional differentiation, and expression patterns of *BBML* genes across plants, laying the groundwork for further investigations into the molecular properties and biological roles of *BBMLs*.

## 1. Introduction

Transcription factors (TFs), serving as a crucial bridge between cell signaling and gene regulation, control organism development, differentiation, and adaptive responses by modulating gene expression in reaction to internal and external signals [1]. TFs can activate or repress the transcription and expression of genes by binding to the specific cis-acting elements in the promoter areas [2]. Several important TF families related to plant growth and development have been identified, for instance, WOX (WUSCHEL-related homebox), WRKY, MADS-box, and DOF (DNA-binding with one finger). BABY BOOM (BBM), a TF in the APETALA2 (AP2) protein family, participates in triggering embryo formation from differentiated somatic cells [3].

Non-zygotic or somatic embryogenesis is a vital technique for clonal propagation and plant transformation, involving cellular reprogramming of differentiated somatic cells to acquire pluripotency [4,5]. Accumulating evidence suggests that ectopic expression of the *BBM* gene is capable of inducing asexual embryo formation [6,7], and this phenomenon has been extensively utilized to enhance clonal propagation and plant transformation. For example, in *Arabidopsis thaliana*, ectopic *BBM* expression in the egg cell has sufficient capacity to bypass the requirement for fertilization and initiate embryo development [6]. The combination of *bbm* and *plt2* mutant causes a range of morphological defects, including irregular cell division planes, arrested zygotes and misshapen cells in the embryo proper [6]. In *Oryza sativa*, ectopic *BBM1* expression can activate downstream *YUC* genes, thus initiating somatic embryogenesis without exogenous auxins [8]. Further combination of ectopically expressed *BBM1* or *BBM4* with *Mitosis instead of Meiosis* (*MiMe*) can engineer synthetic apomixis in rice [9,10,11]. In addition, in *Zea mays*, overexpression of *BBM* and *Wuschel2* (*WUS2*) can significantly increase the proportion of transgenic callus, thereby improving conversion efficiency [12]. Although the functions of *BBML* genes have been well investigated, they still require a more exhaustive exploration of the evolution of their structures and functions across plant species.

Gene duplication is prevalent in plant genomes and plays a crucial role in plant evolution by generating the raw genetic material for adaptation and diversification [13,14]. Multi-gene families that form after gene duplication will experience four possible evolutionary fates, leading to functional diversification: pseudogenization, neofunctionalization, subfunctionalization, and subneofunctionalization [15,16,17]. To date, studies of *BBML* genes have primarily focused on individual species and their functions in somatic embryogenesis. With the growing availability of transcriptome and genomic data, we are now able to study the *BBML* genes from a broader perspective. Angiosperms are not only the most diverse plant group on land but also an important source of human food, such as the soybean and grape in eudicots and rice and corn in monocots. In our research, 24 *BBML* members were detected through a genome-wide analysis of 10 representative angiosperms. The evolutionary processes and functional diversity of *BBMLs* in different plant species were thoroughly examined, such as phylogeny, gene structures, homologous relationships, cis-acting elements, protein–protein interactions, molecular docking, and expression patterns in different tissues. This research offers important insights for better comprehending the phylogenetic evolutionary relationship and functional diversity of *BBMLs* in plants for better application in the apomixis and agricultural production.

## 2. Materials and Methods

### 2.1. Identification of BBM-like Genes in Plant Species

To explore the evolutionary history of *BBM-like* genes, we selected 10 representative species from angiosperms, including one basal species (*Amborella trichopoda*), four monocots (*Acorus tatarinowii*, *Oryza sativa*, *Zea mays*, and *Triticum aestivum*), and five eudicots (*Vitis vinifera*, *Nelumbo nucifera*, *Glycine max*, *Arabidopsis thaliana*, and *Solanum lycopersicum*). Complete genome datasets and annotation files were obtained from public databases (Table S1), mainly from Ensemble Plants (https://plants.ensembl.org/index.html (accessed on 9 August 2024)) [18], Phytozome v13 (https://phytozome.jgi.doe.gov/pz/portal.html (accessed on 9 August 2024)) [19], and CNGB DataBase (https://db.cngb.org/ (accessed on 9 August 2024)) [20]. Next, the AP2 (PF00847) HMM file acquired in Pfam website (http://pfam-legacy.xfam.org/ (accessed on 9 August 2024)) [21] was utilized as a query to search within tested species using HMMER v3.3.2 [22]. Conserved Domain Database (https://ncbi.nlm.nih.gov/cdd (accessed on 9 August 2024)) [23] and SMART website (http://smart.embl-heidelberg.de (accessed on 9 August 2024)) [24] were employed to verify proteins containing either two repeated AP2 domains or an AP2 domain similar to the AP2 domain in the repeated domain group. AP2 proteins were obtained by deleting the genes without the characteristic of the AP2 family and retaining a representative transcript of each gene.

The multiple sequence alignment of identified AP2 proteins was operated by Muscle v5 with the default parameters [25]. FastTree [26] was employed to build the evolutionary tree by maximum-likelihood (ML) method. The resulting treefile was sent to the iTOL (https://itol.embl.de/ (accessed on 11 August 2024)) for further adjustment [27]. The branch of candidate BBML proteins was localized using previously identified and characterized BBM proteins from *Oryza sativa* [7] (GenBank: BBM1, LOC_Os11g19060; BBM2, LOC_Os02g40070; BBM3, LOC_Os01g67410; BBM4, LOC_Os04g42570) and *Arabidopsis thaliana* [6] (GenBank: BBM1, AT5G17430).

Calculations of the number of amino acids (aa), molecular weight (MW), instability coefficient and isoelectric point (pI) of each BBML were performed in the online software ExPASy (https://web.expasy.org/protparam/ (accessed on 12 August 2024)) [28] (Table S2). Plant-mPLoc website tool (http://www.csbio.sjtu.edu.cn/bioinf/plant-multi/ (accessed on 12 August 2024)) [29] was employed for the prediction of the subcellular localization.

### 2.2. Phylogenetic, Conserved Motif, and Gene Structure Analysis of BBMLs

We used the same method mentioned above to perform a phylogenetic analysis for the identified BBML proteins. Then, the fourteen most conserved motifs of BBMLs were identified via MEME (https://meme-suite.org/meme/tools/meme (accessed on 10 August 2024)) [30]. Exon and intron information for the *BBML* members was extracted from the GFF3 annotion files of each species. The above results were visualized by TBtools (v2.117) [31].

### 2.3. Prediction of Cis-Acting Elements and miRNA Binding Sites

The sequences 2000 bp upstream of the start codon of the *BBML* genes were collected through “Gtf/Gff3 Sequences Extract” and “Fasta Extract (Recommended)” in TBtools. The combination of promoters from each BBML was sent to PlantCARE website (http://bioinformatics.psb.ugent.be/webtools/plantcare/html/ (accessed on 12 August 2024)) [32] for cis-acting element analysis (Table S3) and then displayed by TBtools [31]. The dataset of plant miRNA was gained in sRNAanno (http://www.plantsrnas.org/ (accessed on 13 August 2024)) [33], which classifies miRNAs based on their similarity to known miRNAs from other species, labeling highly similar ones as known and those with low similarity as novel. The psRNATarget (v2017) with default parameters [34] was utilized to predict miRNA target sites of *BBML* genes, and the results were visualized with the Chiplot website (https://www.chiplot.online/ (accessed on 15 August 2024)) (Figure S1).

### 2.4. Analysis of Gene Duplication Events and Collinearity

MCScanX [35] was utilized to analyze the gene duplications and explore the putative homologous chrmosomal regions of *BBMLs* with default parameters. TBtools’ simple Ka/Ks calculator was employed to compute the synonymous (Ks) and nonsynonymous (Ka) mutation rates for the *BBML* collinear pairs. Selection pressures acting on these genes were assessed by Ka/Ks ratio (Ka/Ks < 1, purifying; or Ka/Ks > 1, positive) during evolution [36]. Intraspecific and interspecific collinearity links were displayed using TBtools’ Advanced Circos [31].

### 2.5. Construction of Protein–Protein Interaction Networks and Functional Enrichment

To evaluate interactions of BBMLs with other interacting proteins, the sequences of the selected BBML proteins from different species were submitted to STRING (https://string-db.org/ (accessed on 25 August 2024)) [37] with default parameters for constructions of protein–protein interaction (PPI) networks and were visualized with Cytoscape [38] (Figure S2 and Table S6). Interactors for which the only evidence was “co-mentioned in Pubmed abstracts” were excluded from the analysis. The sequences of interacting proteins were acquired from Uniport (https://www.uniprot.org/ (accessed on 26 August 2024)). TBtools [31] was utilized for the computation of the protein pairwise similarity matrix between interacting proteins from different species and constructing the similarity heatmap. Then, Gene Ontology (GO) enrichment analysis was executed with the Gene Ontology website (https://geneontology.org/ (accessed on 26 August 2024)) [39] (Table S7). We focused on biological processes, and GO terms with *p*-value ≤ 0.01 were selected and visualized in a tool website (https://www.bioinformatics.com.cn (accessed on 27 August 2024)) [40].

### 2.6. Three-Dimensional Structure Modeling and Molecular Docking

AlphaFold3 (https://alphafoldserver.com/ (accessed on 3 September 2024)) [41] was utilized to model three-dimensional structures of BBML proteins and selected interacting proteins. ClustalX was utilized for multiple sequence alignment [42], which was visualized with ESPript [43] (Figure S3). Then, PyMOL Molecular Graphics (Version 3.0.3 Schrödinger, LLC, New York, NY, USA) was used in eliminating water molecules and adding hydrogens. The pre-treated BBMLs were used as receptors and interacting proteins as ligands, and the molecular docking between receptors and ligands was carried out through GRAMM (http://gramm.compbio.ku.edu/ (accessed on 5 September 2024)) [44]. Finally, the results of molecular docking were visualized with PyMOL, and the binding energy and interface area were calculated with PDBePISA (https://www.ebi.ac.uk/msd-srv/prot_int/ (accessed on 5 September 2024)) [45] (Table S8).

### 2.7. Expression Pattern Analysis of BBML Genes

Transcriptome datasets of *Arabidopsis thaliana*, *Glycine max*, *Oryza sativa*, *Triticum aestivum*, and *Zea mays* for different tissues were downloaded from the Plant Public RNA-seq database (https://plantrnadb.com/ (accessed on 10 September 2024)) [46]. The expression levels were demonstrated as values of fragments per kilobase of transcripts per million mapped reads (FPKM) of sequencing data (more than 2 biological replicates were represented using mean values) and were visualized with an online platform (https://www.bioinformatics.com.cn (accessed on 12 September 2024)) [40].

## 3. Results

### 3.1. Identification of BBMLs in Multiple Species

**Figure 1:**
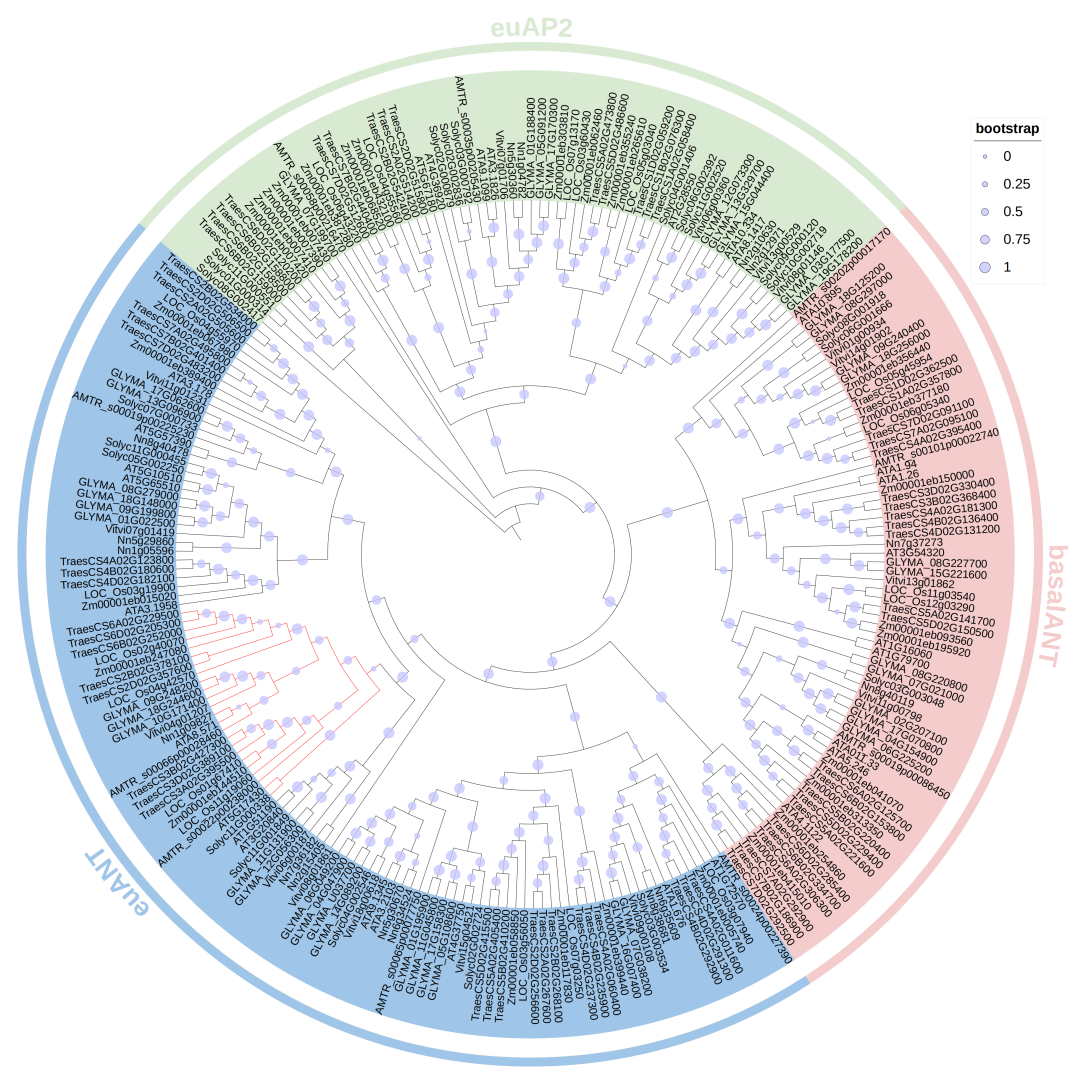
Phylogenetic analysis of BBML proteins from ten tested species. The phylogenetic tree was constructed based on the maximum-likelihood method, divided into three groups that were identified as euAP2, basalANT, and euANT. The branch in red represents putative BBMLs. The circle size indicates the bootstrap value.

Ten representative angiosperms were selected for the phylogenetic analysis of AP2 proteins, including one basal species (*Amborella trichopoda*), four monocots (*Acorus tatarinowii*, *Oryza sativa*, *Zea mays*, and *Triticum aestivum*), and five eudicots (*Vitis vinifera*, *Nelumbo nucifera*, *Glycine max*, *Arabidopsis thaliana*, and *Solanum lycopersicum*). All genomes of the selected species have been well sequenced. To identify putative AP2 proteins, the HMM profile of the AP2 domain (Pfam ID: PF00847) was used as query against the protein dataset of the above species. Subsequently, all putative proteins predicted to encode AP2 domain-containing proteins were used to calculate the number of AP2 domains with the CDD and SMART programs. The analysis identified 249 proteins whose sequence contained two AP2 domains. According to the maximum-likelihood (ML) phylogenetic tree (Figure 1) and subfamily classification of AP2 proteins [47,48], proteins were classified into euAP2, basal-AINTEGUMENTA (basalANT), and BBM-containing euANT subfamilies.

Then, we used defined BBMs from *O. sativa* (Os) and *A. thaliana* (At) to locate the branch of BBMLs, and 24 putative BBMLs were retrieved. The number of BBML proteins varied significantly across the species tested. For example, 8, 3, 2, and 1 BBMLs were detected in *T. aestivum* (Ta), *G. max* (Gm), *Z. mays* (Zm), and *S. lycopersicum* (Sl), while only two genes were identified in basal plant *A. trichopoda*. These data indicated that BBMLs tended to gradually expand or be lost during the evolution process. A detailed summary of the characteristics of the identified BBML proteins in the tested species, including number of amino acids (aa), theoretical isoelectric point (pI), molecular weight (MW), subcellular location, etc., were presented in Table S2.

### 3.2. Phylogenetic, Conserved Motif, and Gene Structure Analysis of BBMLs

**Figure 2:**
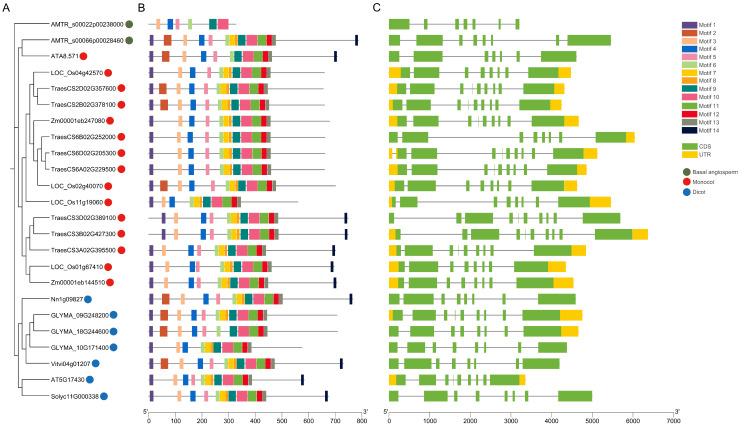
Phylogenetic evolutionary tree, conserved motifs, and gene structures of 24 BBML proteins. (**A**) Phylogenetic tree of BBML proteins. (**B**) Conserved motifs of the BBML proteins. Diverse colors indicate fourteen motifs. (**C**) Structural composition of *BBML* genes. Black lines, yellow boxes, and green boxes represent introns, CDSs, and UTRs, respectively. The scale at the bottom contrasts gene and protein lengths.

Evolutionary relationships among BBML proteins were analyzed by generating a phylogenetic tree. BBMLs from monocots and eudicots were classified into two relatively independent clades (Figure 2A). To investigate the evolutionary and structural diversity of BBMLs, the conserved motifs within BBML proteins were analyzed. Fourteen distinct and highly conserved motifs were named as motif 1 to motif 14 (Figure 2B). Except for motifs 2 and 14, other motifs were present in almost all the members of BBMLs. Among them, motif 4 had a similar sequence to bbm-1 motif [49]. Motifs 6–13 comprised two AP2 domains, along with a linker region in between, and there was a partial loss of AP2 domains in AMTR_s00022p00238000 and GLYMA_18G244600. Moreover, different structures determine the differential function and expression of genes. The intron/exon structures were obtained from GFF3 annotation files to further study the structural diversity of *BBMLs* (Figure 2C). The analysis results indicated that the exon numbers ranged from 7 to 10, and microexons (≤51 nucleotides) were observed in some *BBML* genes, such as *TraesCS2B02G378100*, *TraesCS6A02G229500*, *GLYMA_09G248200*, and *AT5G17430*. In allohexaploid wheat, *BBML* genes from different subgenomes, such as *TraesCS6B02G252000*, *TraesCS3B02G427300*, and *TraesCS3D02G389100*, might have undergone intron insertion or expansion during evolution, potentially affecting the function and expression of genes [50]. Among all branches, 14 genes possessed both 3′ and 5′ untranslated regions (UTRs), whereas *BBMLs* from *A. trichopoda*, *A. tatarinowii*, *N. nucifera*, *V. vinifera*, and *S. lycopersicum* lacked a UTR sequence.

### 3.3. Analysis of Cis-Acting Elements of the BBML Promoter

**Figure 3:**
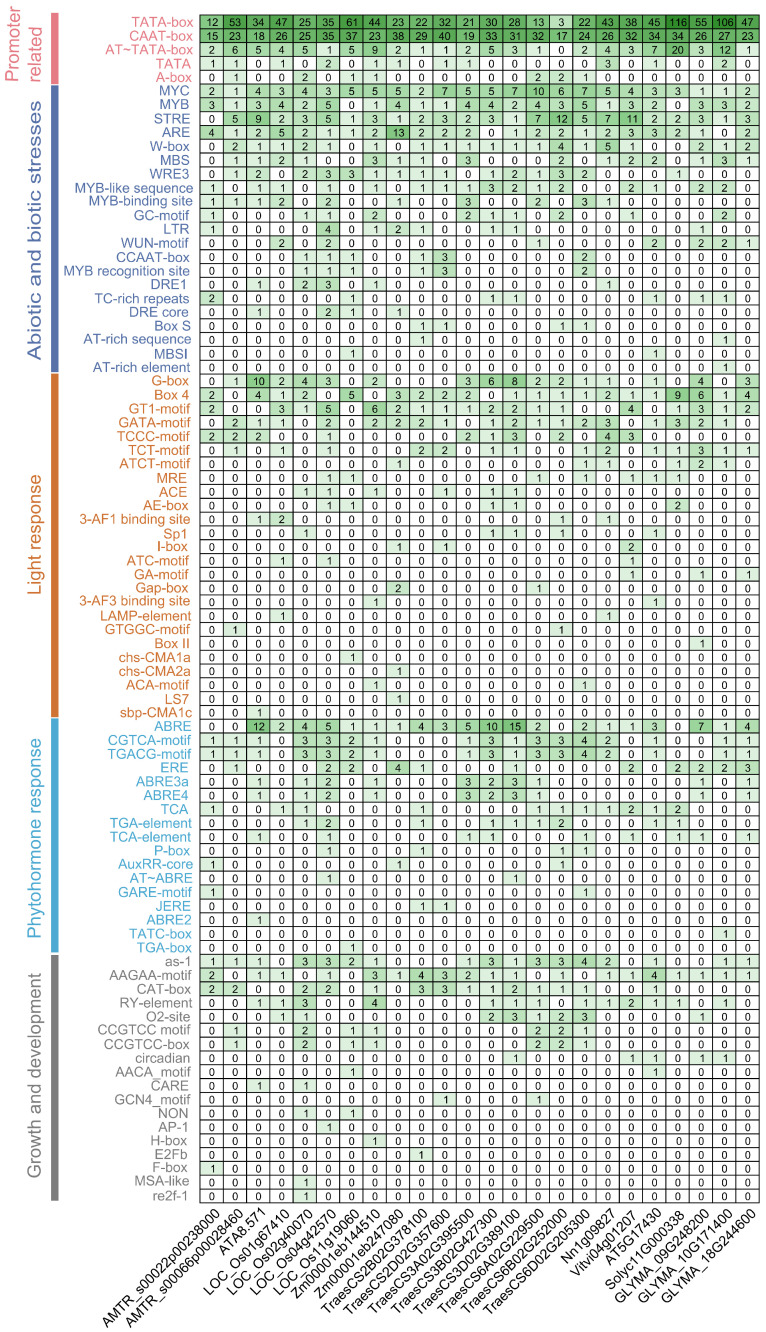
Predicted cis-acting elements of *BBML* genes. The number in each box represents the number of corresponding elements involved in the extracted promoter regions.

Gene expression at the promoter level is primarily regulated by multiple cis-acting elements within the promoter zone [51]. To explore the expression regulation mechanisms of *BBMLs*, the 2000 bp promoter sequences was taken and uploaded to the PlantCARE website for the identification of various cis-acting elements within promoter areas. Ninety cis-acting elements were gained and classified into five groups: (i) promoter-related, (ii) abiotic and biotic stresses, (iii) light response, (iv) phytohormone response, and (v) growth and development (Figure 3 and Table S3). In promoter-related elements, both the TATA-box and CAAT-box were detected to be highly abundant among all *BBMLs*, serving as TF binding sites for transcription initiation. The TATA-box helped bind to the initiation site, while the AT-TATA and A-box functioned as promoter binding sites. In the abiotic and biotic stress-responsive group, such as MYB/MYC binding site elements, ARE and STRE were widely located in the *BBML* promoters. *BBMLs* had a larger variety of elements associated with light response, of which Box 4 and G-box elements took up most of the category. ABRE, also known as abscisic acid-responsive element, was found to be the most numerous phytohormone response element in *BBMLs*. Among the growth and development elements, those located in the promoter zones included the as-1 accountable for root-specific expression, the CAT-box accountable for meristem expression, an RY-element accountable for seed-specific regulation, etc. Moreover, elements with unknown functions like Unnamed_4 and Unnamed_1 were abundant in *BBMLs*, which may had important functions (Table S3). These findings demonstrate that *BBMLs* might perform a vital function in stress resistance and plant development in roots and seeds.

### 3.4. Prediction of miRNA Binding Sites

In plants, miRNAs negatively regulate target genes’ expression by mRNA cleavage or translation repression, serving critical roles in plant growth and secondary metabolism [52]. Previous studies have identified the miR172/AP2 module as a key regulator controlling inflorescence meristem size by regulating the area and number of cells [53]. To determine whether *BBMLs* were regulated by other miRNAs, and the potential miRNA binding sites of the 24 genes were predicted. The results showed that these genes were potentially targeted by 52 known miRNAs (Figure S1). *TraesCS6A02G229500* and *TraesCS6D02G205300* had the highest number of miRNA targets. In monocots, miR167 and miR169 had the most target genes (five each), followed by miR159 (four), while in eudicots, miR156 had the most target genes (five), followed by miR393 (three). In addition, 18 genes were targeted by 75 novel miRNAs (Table S4).

### 3.5. Gene Duplication and Collinearity Analysis of BBML Genes

**Figure 4:**
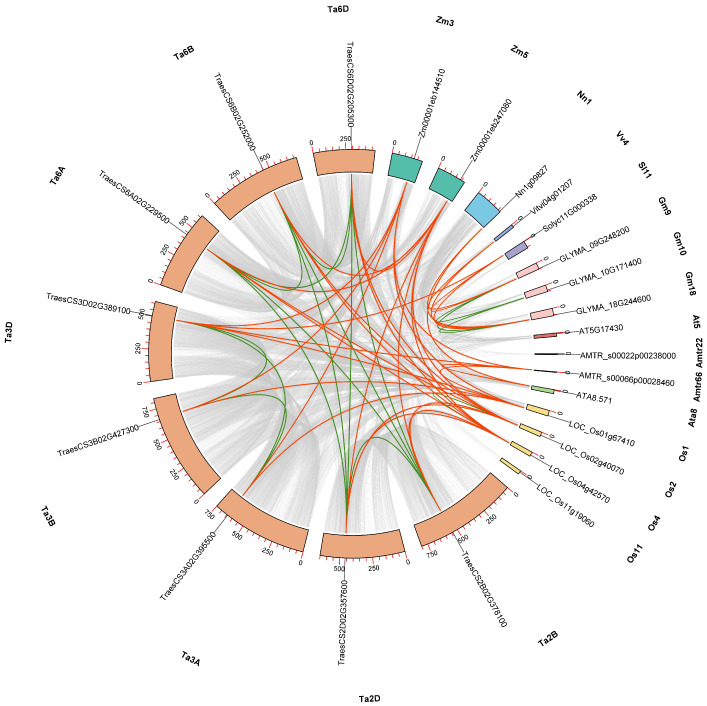
Extensive microcollinearity of BBM gene pairs across tested species. The chromosomes of different plant are represented by distinct colors. Amtr, Ata, Os, Ta, Zm, At, Nn, Vv, Sl, and Gm represent *A. trichopoda*, *A. tatarinowii*, *O. sativa*, *T. aestivum*, *Z. mays*, *A. thaliana*, *N. nucifera*, *V. vinifera*, *S. lycopersicum*, and *G. max*, respectively. The red curved lines denote inter-collinear relationships, and the green line represent intra-collinear relationships, as well as segmental duplication events. The gray lines symbolize the duplication events in other regions. Only the *BBML*-containing chromosomes were included.

Homologous genes are primarily categorized into two groups: orthologous and paralogous. Gene family expansion or evolution is primarily driven by segmental and tandem duplications [54]. To further explore the expansion/contraction mechanisms and the homology of *BBMLs*, synteny and gene duplication events of *BBML* genes from 10 tested species were analyzed using MCScanX (Figure 4).

A total of 52 collinear gene pairs were detected, including 17 intra-collinear pairs and 35 inter-collinear pairs. Although the number of intra-collinear pairs varied greatly among these 10 species, all of them were identified as segmental duplication events, demonstrating that the amplification mechanism of *BBML* genes was relatively conserved (Table 1). These findings suggested that gene duplications, particularly segmental duplications, performed a crucial role in the amplification of *BBMLs* in the tested species.

To further study the orthologous relationships and duplication events of *BBMLs* among the 10 tested species, total of 35 interspecies collinear gene pairs were identified through genome comparison (Figure 4 and Table S5). Among the monocots, 13 collinear gene pairs were detected between *T. aestivum* and *O. sativa*, 8 pairs between *T. aestivum* and *Z. mays*, and 3 pairs between *O. sativa* and *Z. mays*. Although no inter-collinear pairs could be observed between *ATA8.571* and other genes, collinearity was detected with regions near *LOC_Os01g67410*, *Zm00001eb144510*, *TraesCS3A02G395500*, *TraesCS3B02G427300*, and *TraesCS3D02G389100*. Among the eudicots, two, two, and one collinear gene pairs were severally found between *G.max* and *V. vinifera*, *S. lycopersicum*, and *N. nucifera*. No inter-collinear pairs could observed for *AT5G17430* and *GLYMA_10G171400*. In addition, inter-collinear pairs were observed between *AMTR_s00066p00028460* from basal angiosperm *A. trichopoda* and *ATA8.571*, *LOC_Os01g67410*, *TraesCS3D02G389100*, and *Zm00001eb144510* from monocots. There was no collinearity between *A. trichopoda* and eudicots, suggesting a distinct genomic structure and functional variation during the evolution.

Furthermore, to better understand the evolutionary process, the value of synonymous (Ks) and non-synonymous (Ka) substitution of *BBML* collinear pair was calculated, and Ka/Ks were used to assess the effectiveness of evolutionary constraints. The calculation results demonstrated that, except for two duplicated pairs which showed NaN because of high sequence divergence value, all the other duplicated pairs showed Ka/Ks < 1, varying from 0.0588 to 0.3811, suggesting that all duplicated gene pairs have undergone purifying selection throughout evolution (Table 1 and Table S5).

### 3.6. Construction of Protein–Protein Interaction Networks and Functional Enrichment

**Figure 5:**
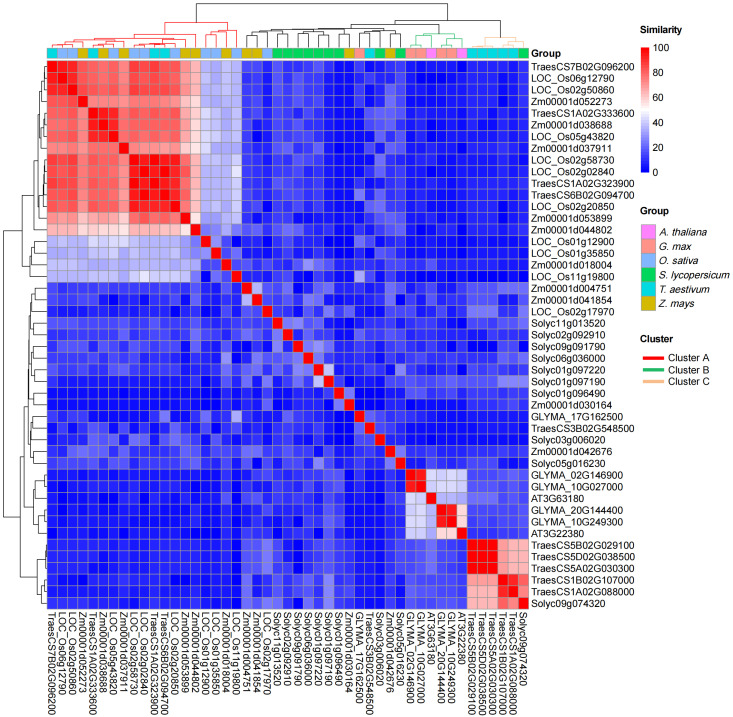
The heatmap of the sequence similarity of interacting proteins from *A. thaliana*, *G. max*, *O. sativa*, *S. lycopersicum*, *T. aestivum*, and *Z. mays*.

To further explore the function of BBML proteins in the tested species, we constructed the protein–protein interaction (PPI) network for BBMLs from *O. sativa*, *Z. mays*, *T. aestivum*, *A. thaliana*, *S. lycopersicum*, and *G. max* using the STRING website tools (Figure S2). The annotation information of interacting proteins was obtained from NCBI and Phytozome (Table S6). The interaction network showed that some interacting proteins also had interactions between each other, suggesting that they might play a pivotal role in the network, such as *O. sativa* (LOC_Os02g17970) (Figure S2A), *Z. mays* (Zm00001d042676 and Zm00001d030164) (Figure S2B). and *S. lycopersicum* (Solyc09g091790 and Solyc01g096490) (Figure S2E). A pairwise similarity matrix was also constructed and visualized to explore whether BBMLs from the tested species function in similar or different pathways (Figure 5). In the matrix, interacting proteins formed three clusters of high similarity, clusters A to C. Cluster A was specific to monocots, cluster B was specific to eudicots, and cluster C was specific to *T. aestivum* but contained one protein from *S. lycopersicum*. Further studies showed that the proteins in cluster A were both from the Ras superfamily, with the majority belonging to the Rho/Rac GTPase subfamily, which had been found to be involved in cell polarity, cell shape, hormone responses, and pathogen defense [55,56,57]. The proteins in cluster B were both described as TIC-like proteins and the proteins in cluster C as PSTP (protein serine/threonine phosphatase).

**Figure 6:**
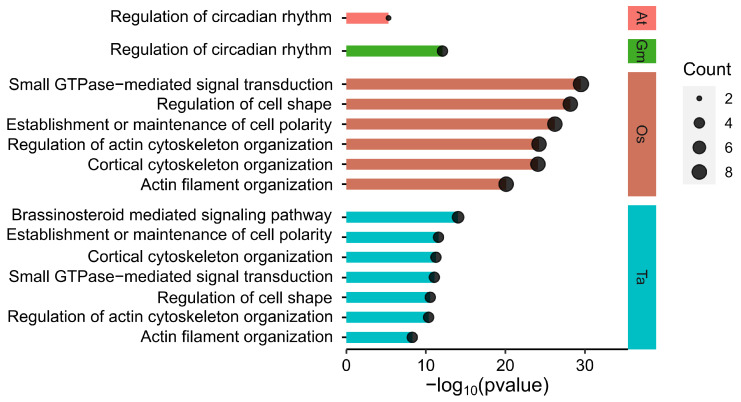
Gene ontology (GO) enrichment of interacting proteins in *A. thaliana*, *G. max*, *O.sativa*, and *T. aestivum*.

To explore the biological processes that these interacting proteins may participate in, we then conducted functional enrichment analysis of interacting proteins from *A. thaliana*, *G. max*, *T. aestivum*, and *O. sativa* (Figure 6 and Table S7). The significantly enriched gene ontology (GO) terms showed that interacting proteins in *A. thaliana* and *G. max* were mainly described as being involved the regulation of circadian rhythm, while interactors in *O. sativa* and *T. aestivum* were mainly involved in the establishment or maintenance of cell polarity, small GTPase-mediated signal transduction, regulation of cell shape, etc. These results suggest that there might be distinctions in BBML functions between monocots and eudicots.

### 3.7. Three-Dimensional Structure Modeling and Molecular Docking

**Figure 7:**
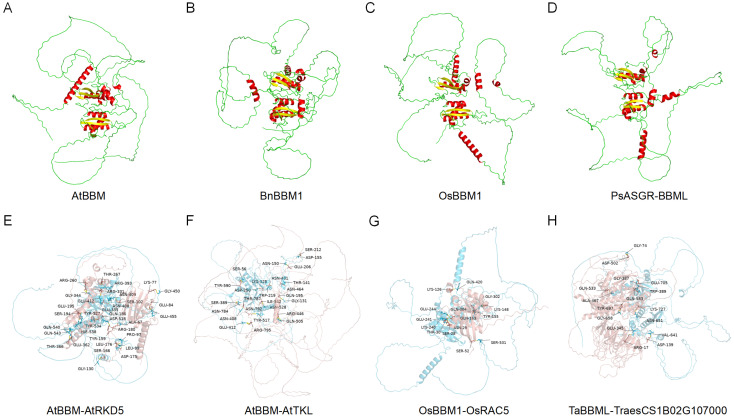
Three-dimensional structure modeling and molecular docking of BBML proteins. (**A**–**D**) Three-dimensional protein structure of AtBBM (**A**), BnBBM1 (**B**), OsBBM1 (**C**), and PsASGR-BBML (**D**). Red symbolizes α-helix, yellow symbolizes β-fold, and green symbolizes irregular curl. (**E**,**F**) The receptor–ligand interaction of interacting proteins with BBML active sites. The blue and red colors symbolize the receptor and ligand, respectively. (**E**) Molecular docking of AtBBM with AtRKD5, (**F**) molecular docking of AtBBM with AtTKL, (**G**) molecular docking of OsBBM1 with OsRAC5, and (**H**) molecular docking of TaBBM with TraesCS1B02G107000.

The protein’s function is determined by its 3D space structure, which in turn depends on its amino acid sequence. To explore the structural characteristics of BBML proteins and their relationship with parthenogenetic induction function, we selected one BBM homologous protein from each species of *A. thaliana* (At), *Brassica napus* (Bn), *O. sativa* (Os), and *Pennisetum squalatum* (Ps). The parthenogenetic induction ability of the selected proteins has been tested [6,7,58]. Then, we predicted the three-dimensional protein model using AlphaFold3 (Figure 7A–D). The result showed that the AtBBM (AT5G17430), BnBBM1 (AF317904), OsBBM1 (LOC_Os11g19060), and PsASGR-BBML (EU559280) proteins exhibited similar structures across different species, such as α-helices (α1–6), $3_{10}$-helices (η1–4), and β-strands (β1–6) (Figure S3). These highly conserved structures might have an important biological function. In addition, special structures αA and αC were found in OsBBM1 and PsASGR-BBML, while αB and αD were found in AtBBM and BnBBM1. These special structures were likely related to the functional differences of BBML in eudicots and monocots. Further studies found that the location of helix αD overlapped with the binding sites of *AtBBM* and its inhibitory factor AtRKD5 in *Arabidopsis* [59], indicating a potential involvement in the repression of AtBBM expression in the egg cell. Next, we selected AtBBM (AT5G17430) with inhibitory factor AtRKD5 (AT4G35590), OsBBM1 (LOC_Os11g19060) with OsRAC5 (LOC_Os02g58730) from cluster A, AtBBM (AT5G17430) with AtTKL (AT3G63180) from cluster B, and TaBBML (TraesCS2B02G378100) with TraesCS1B02G107000 from cluster C to further predict the binding sites between BBMLs and putative interacting proteins with AlphaFold3 and GRAMM (Figure 7E–H). BBMLs were used as receptors and interacting proteins as ligands for molecular docking. The proteins were connected by multiple pairs of amino acid residues through chemical bonds, mainly through hydrogen bonds. More detailed information on the molecular docking for each group, including binding energy, interface area, and hydrogen bonds, is presented in Table S8, which could provide more references for the application of BBMLs.

### 3.8. Expression Patterns of BBMLs in Plants

**Figure 8:**
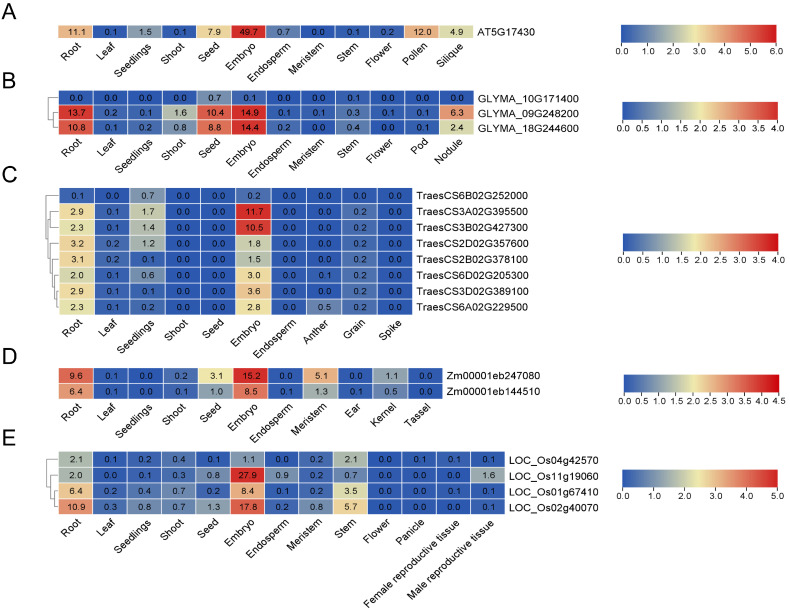
The expression heatmap of *BBMLs* based on the RNA-seq database in various tissues from representative species, including *A. thaliana* (**A**), *G. max* (**B**), *T. aestivum* (**C**), *Z. mays* (**D**), *O. sative* (**E**), respectively. The values in each box represent the relative expression levels.

Expression patterns offer valuable perspectives into the transcriptional divergence of genes [60]. To further investigate the potential biological functions of the *BBML* genes, transcriptional data of *BBMLs* in various tissues and organs were analyzed based on the PPRD RNA-seq database. In *A. thaliana* (Figure 8A), *AT5G17430* exhibited high expression level in the embryo and also demonstrated elevated expression in the root, pollen, silique, and seed. *AT5G17430* was barely expressed in other tissues, such as the shoot, leaf and flower. In *G. max* (Figure 8B), among the three genes that form segmental duplicated gene pairs, *GLYMA_10G171400* was barely expressed in all tissues. *GLYMA_09G248200* and *GLYMA_18G244600* exhibited similar expression patterns, which showed high expression across various tissues, such as root, seed, embryo, and nodule. In *T. aestivum* (Figure 8C), *TreasCS6B02G252000* was found to be absent in almost all tissues, which may indicate functional redundancy. Among the remaining genes, all were expressed in the embryo and root, and *TraesCS3A02G395500*, *TraesCS3B02G427300* and *TraesCS3D02G389100* also had a low expression in seedlings. In *Z. mays* (Figure 8D), *Zm00001eb144510* and *Zm00001eb247080* exhibited similar expression patterns across most tissues. For instance, both of them showed high expression in the embryo and root, with *Zm00001eb247080* displaying relatively higher expression levels. In *O. sativa* (Figure 8E), *LOC_Os11g19060* was expressed in a limited number of tissues, with high specificity in the embryo, while its expression levels were lower in the root and male reproductive tissue. Compared with *LOC_Os11g19060*, *LOC_Os01g67410* and *LOC_Os02g40070* were not only expressed in the root or embryo but also showed high expression levels in the stem. Interestingly, *LOC_Os04g42570*, which belongs to the segmental duplication pair of *LOC_Os02g40070*, had very low expression levels in the root, stem, and embryo. Overall, the *BBML* genes were mainly expressed in embryo and root tissues, with a significant tissue-specific expression pattern, which implies their possible role in embryo and root development.

## 4. Discussion

Apomixis, a progress of asexual reproduction that generates clonal seeds with heterozygous genotypes, can be induced by combining *MiMe* (*Mitosis instead of Meiosis*) with a mutant of gene *MATRILINEAL* [61,62] or *DMP* [63] or through ectopic expression of gene *WUSCHEL* [64] or *BBM* [11] in the egg cell. BABY BOOM (BBM) is a member of the APETALA2/ETHYLENE RESPONSE FACTOR (AP2/ERF) family that plays a crucial role in the regulation of plant cell totipotency, as it triggers asexual embryo formation when ectopically expressed [65]. Genome-wide BBM-like TF identification studies have been conducted in *A. thaliana* [3], *O. sativa* [7], *Z. mays* [66], and other plants. Many studies aimed to study the capability of *BBMLs* in somatic embryogenesis and parthenogenesis across different species [6,7,58]. Nevertheless, the dynamical evolutionary process and functional differentiation of plant *BBML* genes have received limited attention. With the recent availability of the complete genome sequences from various plant species, comprehensive genome-wide surveys and molecular evolution studies can now be conducted to study the plant *BBML* genes. We executed a extensive genome-wide survey of AP2 family members from 10 species and located the branch of BBMLs (Figure 1). We constructed the maximum-likelihood (ML) tree of BBML proteins from one basal angiosperm, four monocots, and four eudicots to study the phylogenetic relationships between BBMLs (Figure 2). Next, we identified the motif compositions, gene structures, cis-acting elements, binding microRNAs, and transcriptional factors and conducted collinearity, protein–protein interaction network, three-dimensional modeling, and molecular docking analyses.

The highly conserved motifs and domains contained in TFs always play an important role in their regulatory activities [67]. In conserved motif analysis, eight motifs (motifs 6–13) combined into the AP2 domain and were highly conserved in all BBMLs. Notably, motif 4 (bbm-1 motif) was found to be present in all putative BBML proteins, which was verified to be specific to BBMLs and was essential in regulating somatic embryogenesis and embryo development [49]. These conserved structural elements probably indicate the important roles that BBMLs play in different kinds of physical DNA binding. Gene structure analysis observed microexons (≤51 nucleotides) in some *BBML* genes, which widely exist in genes for plant development and environmental responses [68]. Gene expression is primarily regulated by upstream promoters. The specific binding of transcription factors to cis-acting elements in the promoter region is crucial for biological signal transduction and also plays a key role in synergy with other genes [69]. Through the investigation of cis-acting elements (Figure 3), a sum of 90 elements located in the promoter regions were observed from the 2 kb promoter region of *BBML* genes. In addition to cis-acting elements associated with basic functions (such as site-binding and promoter elements), the *BBML* genes also contained numerous elements linked to abiotic and biotic stresses, as well as light response. This suggested their involvement in light and stress response functions.

Researchers have reported that during evolution, it is common for gene family members to undergo duplication, loss, pseudogenization, and other changes [70]. The number of BBML gene copies varies significantly across different plant species, ranging from only one copy in *A. thaliana*, *S. lycopersicum* and *A. tatarinowii* to eight copies in *T. aestivum* (Figure 2 and Table S2). Gene duplication served an essential function in the expansion of *BBMLs* in certain plant species, leading to differences in the quantity of *BBML* genes among the tested plants. Our analysis identified 13 *BBML* genes within the genome duplication region, resulting from 19 segmental duplicate events. This suggested that the expansion of *BBML* genes in these species was primarily driven by segmental duplication events (Table 1). Compared to other plant species, the *BBML* genes in *T. aestivum* exhibited a significantly higher number of duplication events, which may have resulted from the fusion of three diploid genomes. This event finally led to a substantial increase in duplicated genes within the genome, with the majority containing at least three functional copies [71]. In addition, the Ka/Ks ratio demonstrated that the *BBML* genes were greatly influenced by purification selection during evolution, suggesting their essential roles in plant development and that alterations in their function may negatively affect plant fitness (Table 1 and Table S4).

Previous studies have found that BBM can transcriptionally regulate auxin biosynthesis gene *YUCCA* during somatic embryogenesis in *A. thaliana* [72] and *O. sativa* [8]. Through the String database, a network of BBML proteins and other potential interacting proteins was constructed (Figure S2), including not only the interactions between BBML proteins and known functional proteins, such as RHO protein, which regulates cell division and differentiation [73], and TIC-LIKE protein, a protein transport complex on the chloroplast membrane [74], but also some proteins whose functions have not been verified. These interacting proteins may assist in plant embryogenesis and regulation of cell totipotency. We also used AlphaFold3 for 3D structure prediction to explore the structure characteristics of BBMLs. Most of the structures were conserved between BBML proteins, and we found a special α-helix in AtBBM, which overlapped with the binding sites of *AtBBM* and its inhibitory factor AtRKD5 in *Arabidopsis* [59], indicating a potential involvement in the repression of *AtBBM* expression in the egg cell. Further confirmation can be made by yeast one-hybrid and two-hybrid assays to ensure the relationship between proteins and DNA or proteins. The molecular docking revealed that BBML proteins form chemical bonds with other interacting proteins through specific amino acid residues, such as hydrogen bonds, and thus involved in regulating plant development (Figure 7A–D and Table S8).

Expression analysis of the published transcriptome data from different tissues in the selected species revealed the *BBMLs*’ remarkable tissue specificity, especially their high expression in embryo and root (Figure 8). This discovery aligns with the known expression pattern in the embryo and seed [3,6], as well as the role of *BBML* genes in somatic embryogenesis and embryonic development [6]. In addition, this tissue-specific expression pattern may reflect the fine regulatory mechanism of *BBML* genes in regulating plant development. In the future, qRT-PCR experiments are needed to further validate the expression pattern results obtained from transcriptome data analysis. Overall, our research performed an extensive genomic and phylogenetic analysis of the *BBML* genes in multiple species, revealing the evolutionary dynamics, expression specificity, and potential regulatory mechanisms of the *BBMLs* in different plants, providing valuable basic information and new ideas for the future application of *BBML* genes in crop improvement and plant development research.

## 5. Conclusions

This research examined the phylogenetic classification and characteristic of 24 *BBMLs* among 10 tested species. Conservation and diversification of *BBML* genes were observed through the comprehensive genome-wide studies of phylogenetic relationships, genome structures, duplication events, protein–protein interaction networks, three-dimensional structure, cis-acting elements, molecular docking, and expression patterns. The expansion of the *BBMLs* mainly occurred via segmental duplication events and undergoing strong purification selection. Published transcriptome data analysis demonstrated tissue-specific expression patterns of *BBMLs*, indicating their capability in the plant development of roots and embryos. The molecular docking between BBML proteins and interacting proteins was carried out, and potential functional amino acid residues with junctions between proteins were found. Our findings offer a valuable and thorough reference framework for future research and application of *BBML* genes.

## Figures and Tables

**Table 1:** Ka/Ks analysis for the duplicated *BBML* paralogs in tested species.

Duplicated Gene 1	Duplicated Gene 2	Ka	Ks	Ka/Ks	Duplication Type	Purifying Selection
LOC_Os04g42570	LOC_Os02g40070	0.1735	0.4552	0.3811	Segmental	YES
TraesCS2B02G378100	TraesCS2D02G357600	0.0068	0.0702	0.0961	Segmental	YES
TraesCS2B02G378100	TraesCS6A02G229500	0.1843	0.7305	0.2522	Segmental	YES
TraesCS2B02G378100	TraesCS6B02G252000	0.1792	0.7233	0.2478	Segmental	YES
TraesCS2B02G378100	TraesCS6D02G205300	0.1929	0.7445	0.2591	Segmental	YES
TraesCS2D02G357600	TraesCS6A02G229500	0.1837	0.6973	0.2635	Segmental	YES
TraesCS2D02G357600	TraesCS6B02G252000	0.1776	0.6933	0.2561	Segmental	YES
TraesCS2D02G357600	TraesCS6D02G205300	0.1919	0.7280	0.2636	Segmental	YES
TraesCS3A02G395500	TraesCS3B02G427300	0.0156	0.1884	0.0828	Segmental	YES
TraesCS3A02G395500	TraesCS3D02G389100	0.0133	0.1799	0.0741	Segmental	YES
TraesCS3B02G427300	TraesCS3D02G389100	0.0065	0.1111	0.0589	Segmental	YES
TraesCS6A02G229500	TraesCS6B02G252000	0.0223	0.0863	0.2588	Segmental	YES
TraesCS6A02G229500	TraesCS6D02G205300	0.0195	0.0883	0.2208	Segmental	YES
TraesCS6B02G252000	TraesCS6D02G205300	0.0243	0.0857	0.2835	Segmental	YES
GLYMA_10G171400	GLYMA_09G248200	0.2310	0.7945	0.2907	Segmental	YES
GLYMA_10G171400	GLYMA_18G244600	0.2462	0.8906	0.2765	Segmental	YES
GLYMA_18G244600	GLYMA_09G248200	0.0485	0.1794	0.2703	Segmental	YES

## Data Availability

All data are displayed in the manuscript and Supplementary Materials.

## Supplementary Materials

The following supporting information can be downloaded at: https://www.mdpi.com/article/10.3390/genes15121614/s1, Figure S1: Known miRNA target gene prediction analysis. The targeting of known miRNAs is indicated by different colored lines; Figure S2: Protein-protein interaction (PPI) networks of BBML proteins; Figure S3: Amino acid sequence and secondary structure alignment of AtBBM, BnBBM1, OsBBM1, PsASGR-BBML; Table S1: 10 representative green plants, genome datasets sources and their version imformation used in the present study; Table S2: Characteristic features of 24 *BBML* genes in tested species; Table S3: Cis-acting elements existed in the 2 kb upstream region of *BBML* genes; Table S4: Novel miRNA target gene prediction analysis of *BBML* genes; Table S5: Ka/Ks analysis for the duplicated *BBML* orthologs in tested species; Table S6: Information of interacting proteins in PPI networks; Table S7: Gene ontology enrichment of interacting proteins; Table S8: Results of molecular docking.
