# Supplementary material for: Genome-Wide Identification and Evolutionary Analysis of Functional BBM-like Genes in Plant Species
Source: Genes (Basel). 2024 Dec 17;15(12):1614. doi: 10.3390/genes15121614 (PMC11675363; doi:10.3390/genes15121614)
Supplement: Supplementary file 1 [file genes-15-01614-s001.zip › Supplementary Files/Figure S1.pdf]

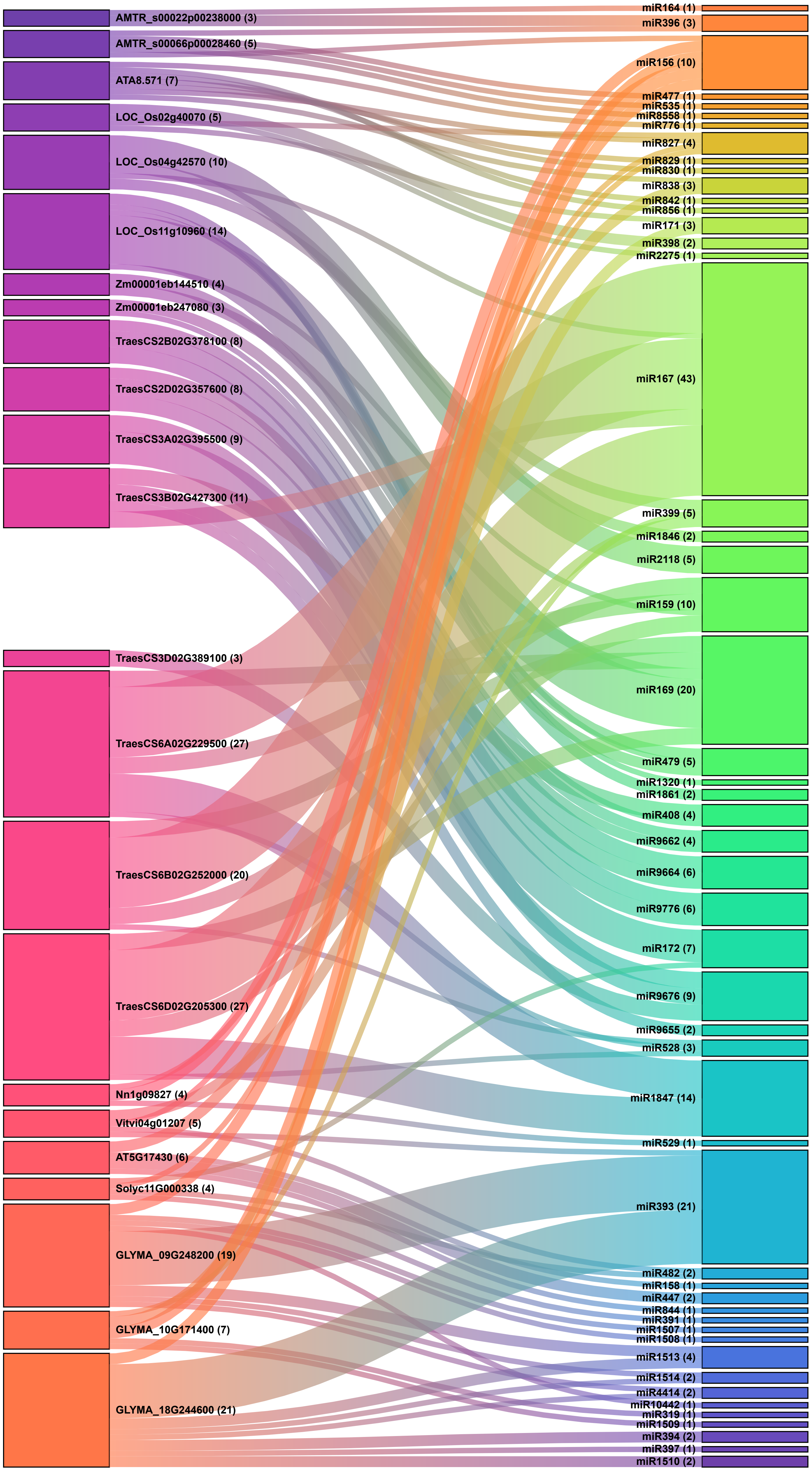

Figure S1. Known miRNA target gene prediction analysis. The targeting of known miRNAs is indicated by different colored lines.
