# Supplementary material for: Genome-Wide Identification and Evolutionary Analysis of Functional BBM-like Genes in Plant Species
Source: Genes (Basel). 2024 Dec 17;15(12):1614. doi: 10.3390/genes15121614 (PMC11675363; doi:10.3390/genes15121614)
Supplement: Supplementary file 1 [file genes-15-01614-s001.zip › Supplementary Files/Figure S2.pdf]

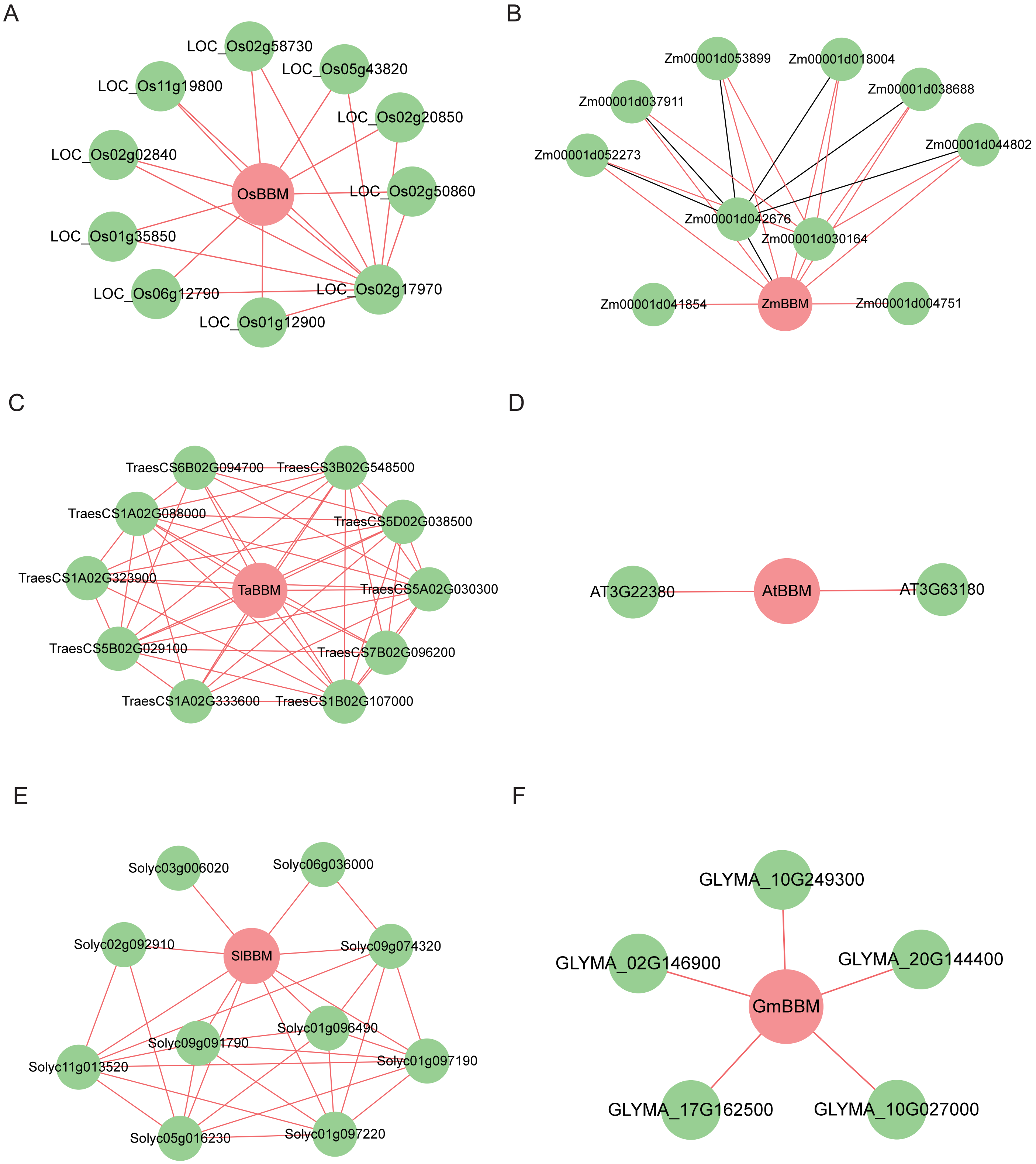

Figure S2. Protein-protein interaction (PPI) networks of BBML proteins. The BBMLs in pink dots represent BBMLs from different species. The green dots are other proteins added based on the string database. The pink line represents experimentally comfirmed interaction relationships and the black line represents predicted interaction relationships. (**A**) PPIs of BBMLs in *O. sative*, (**B**) PPIs of BBMLs in *Z. mays*, (**C**) PPIs of BBMLs in *T. aestivum*, (**D**) PPIs of BBMLs in *A. thaliana*, (**E**) PPIs of BBMLs in *S. lycopersicum*, (**F**) PPIs of BBMLs in *G. max*.
