# Supplementary material for: Genome-Wide Identification and Evolutionary Analysis of Functional BBM-like Genes in Plant Species
Source: Genes (Basel). 2024 Dec 17;15(12):1614. doi: 10.3390/genes15121614 (PMC11675363; doi:10.3390/genes15121614)
Supplement: Supplementary file 1 [file genes-15-01614-s001.zip › Supplementary Files/Figure S3.pdf]

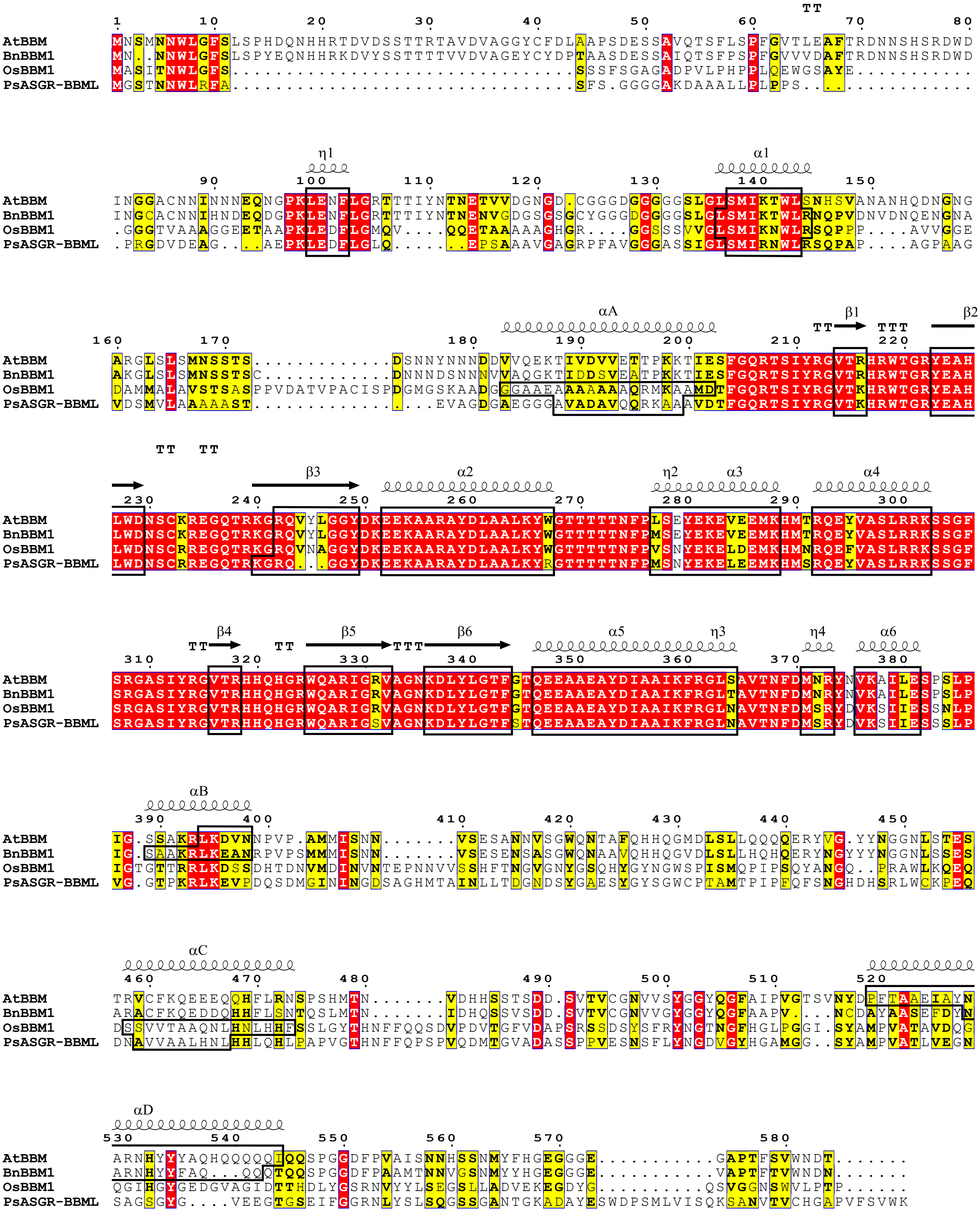

Figure S3. Amino acid sequence and secondary structure alignment of AtBBM, BnBBM1, OsBBM1, PsASGR-BBML. Red background shows sequence identity and yellow background shows sequence similarity in the alignment. The symbol α (α-helices) and η (3_{10}-helices) are displayed as squiggles. β (β-strands) are rendered as arrows, strict β-turns as TT letters and strict α-turns as TTT. Structures common to all proteins are named by numbers, otherwise by letters. Black boxes indicate the sequence of the secondary structure in different proteins.
